# Risk perception towards healthcare waste among community people in Kathmandu, Nepal

**DOI:** 10.1371/journal.pone.0230960

**Published:** 2020-03-30

**Authors:** Sulata Karki, Surya Raj Niraula, Deepak Kumar Yadav, Avaniendra Chakravartty, Sabita Karki

**Affiliations:** 1 School of Public Health and Community Medicine, B.P. Koirala Institute of Health Sciences, Dharan, Nepal; 2 National Academy of Medical Science, Bir Hospital, Kathmandu, Nepal; Imperial College London, UNITED KINGDOM

## Abstract

**Background:**

Healthcare waste management is a serious issue in context of developing countries. Better assessment of both risks and effects of exposure would permit improvements in the management of healthcare waste. However, there is not yet clear understanding of risks, and as consequences, inadequate management practices are often implemented.

**Objectives:**

This study primarily aims to assess risk perception towards healthcare waste and secondly to assess knowledge, attitude and identify the factors associated with risk perception.

**Results:**

A cross-sectional community based study was carried out among 270 respondents selected through multistage sampling technique. Face-to-face interview was conducted using semi-structured questionnaires. Risk perception was classified as good and poor based on mean score. Bivariate and multivariate analyses were carried out to determine the associates of risk perception.

More than half, 52% of the sampled population had a poor risk perception towards healthcare waste. More than a quarter 26.3% had inadequate knowledge and forty percent (40%) had a negative attitude towards health care waste management. Having knowledge (OR = 3.31; CI = 1.67–6.58) was a strong predictor of risk perception towards healthcare waste.

The perception of risk towards healthcare waste among community people was poor. This highlights the need for extensive awareness programs. Promoting knowledge on healthcare waste is a way to change the perception in Nepal. Community engaged research approach is needed to address environmental health concerns among public residents.

## Introduction

The health care industry creates waste materials which, if not properly treated or disposed, can be hazardous to the environment and health of the exposed community. Healthcare industry, with the aim of treating sick people and reducing health problems, inescapably creating waste that is precarious to health. [1] Globally, healthcare waste is second most dangerous waste after radiation waste. [2] Inadequate materials and financial resources, poorly educated human resources and poor governance have contributed to the mismanagement of waste. [3]

Compared to developed countries the risk perception towards wastes generated by the health care industry is much lower in developing countries. [4] Differing views and perceptions have been observed regarding the process of waste generation, segregation, collection, transportation, storage, treatment and disposal. The situation is more critical in the area of planning and resource allocations. [5]

WHO estimated that in 2000, injections with contaminated syringes caused 21 million hepatitis B, 2 million hepatitis C and 260,000 HIV infections. [6] Health care workers and solid waste workers have a higher risk of injury and infection compared to general population. [7] In many low income countries where illegal dumping of healthcare wastes is commonly practiced, children are at high risk of exposure to blood borne viruses. [8] Open burning and inadequate incineration of medical waste is still practiced causing adverse health effects due to the release of highly toxic fumes, as well as contributing to global warming. [9]

Health care waste management has received only intermittent attention in Nepal. [10] Many institutions are dumping waste on the back yard, ditches, rivers, corners of hospital buildings, nearby ponds or anywhere around the premises. Thus, proper management of infectious and hazardous wastes will greatly reduce the risks to public health. [11] Better assessment of both risks and effects of exposure would permit improvements in the management of health-care waste. [12] Even at some healthcare institutions, where some degree of segregation is practiced, all the segregated wastes finally end up in the municipal container. [13] Proper management of healthcare waste has been a major challenge in Nepal, especially in Kathmandu. [14] Therefore, this study examined the risk perception from the community perspective, so that hospitals might give more attention before disposing the wastes which would ultimately improve the management of healthcare waste.

## Methods

### Study design and settings

A cross-sectional community based study was conducted amongst 270 respondents residing near hospitals of Kathmandu. Data was collected from September 1 to November 30, 2017.

### Sampling technique and sample size

**Fig 1:**
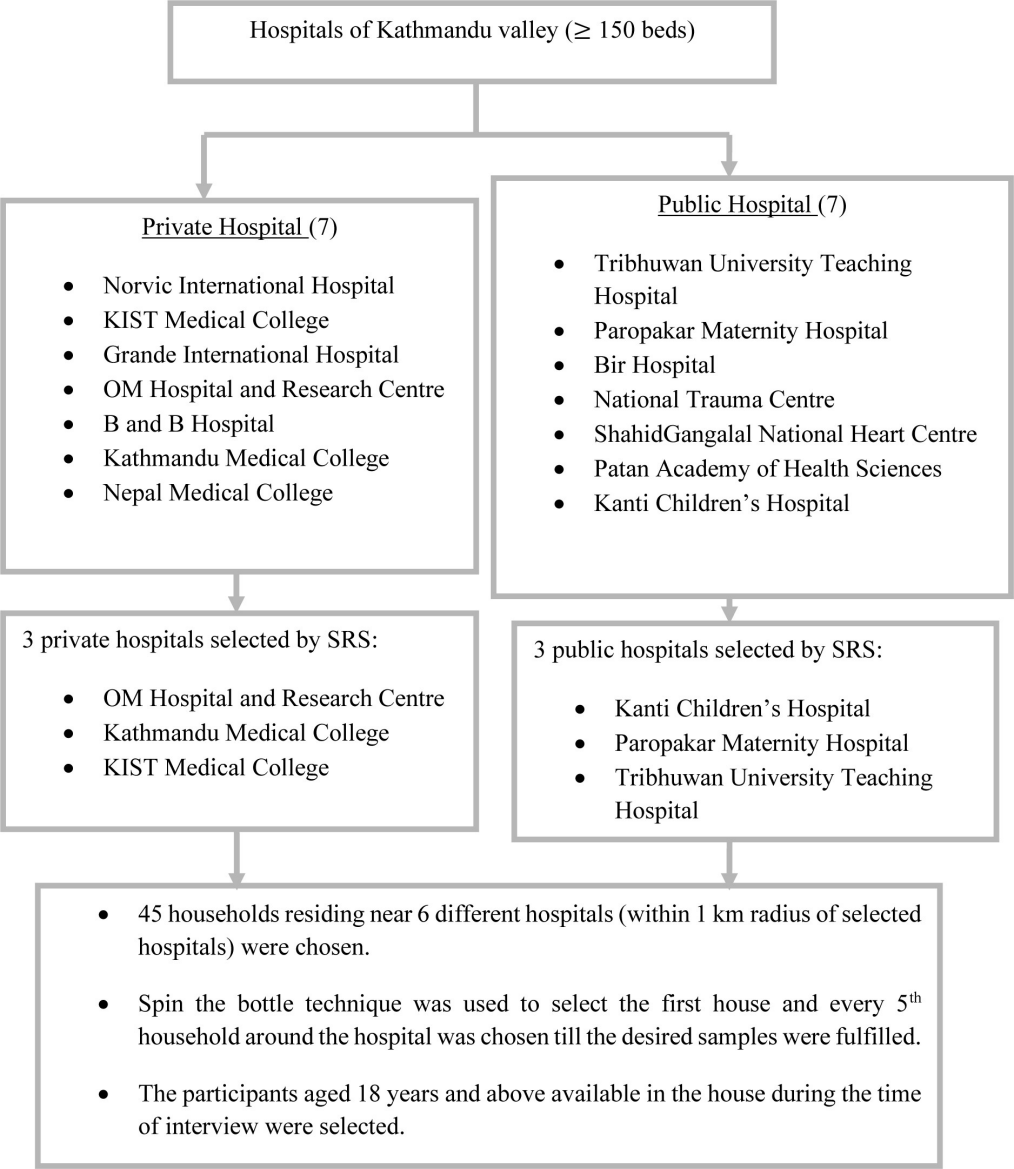
Sampling technique. SRS: Simple Random Sampling.

Kathmandu has a large number of hospitals with tertiary treatment facilities. [15] Based on the data from Department of Health Services 2017, there were a total 14 public and private hospitals with 150 beds and above. Hospitals with 150 beds and above, households residing within 1km distance around these hospitals, and participants aged 18 years and above who consent to participate were the inclusion criteria for the study. Simple random sampling technique was used to select the hospitals, and there after households were selected using systematic sampling (Fig 1).

### Estimation of sample size

Sample size was calculated as per the study by Tadesse Alemayehu et al, which reported 27.2% respondents perceived healthcare wastes as high risk waste. [8] This study considered 95% confidence interval and 80% power for sample size calculation. So, prevalence (p) = 27.2%, compliment of prevalence (q) = 100–27.2 = 72.8%, d = 20% of p = 5.44. Using formula, n = z^2^pq/d^2^ where z = 1.96 at 95% confidence interval and putting all the values in the formula, n = 1.96*1.96*27.2*72.8/29.59 = 257 and adding 5% non-response rate, the final sample size calculated was 270.

### Sampling technique

#### Data collection and analysis

A semi-structured, self-designed questionnaire was developed by reviewing literature. Face to face interview was conducted for the data collection. The questionnaire was finalized in English and then translated into Nepali before the data collection using the translation-back translation method. The interview was conducted in Nepali language. Pretesting of the tool was done by administering the tool to 10% of the total sample in a similar setting. Collected data were entered in Microsoft Excel 2007. The internal consistency was measured via Cronbach’s alpha, which was found to be 0.74 for the Likert scale questions. Microsoft excel sheet was exported into SPSS version 21.0. Binary logistic regression was performed to estimate Odds Ratio with 95% confidence interval.

#### Ethical approval

Ethical approval was obtained from the Institutional Review Committee of B.P. Koirala Institute of Health Sciences (Ref: IRC/145/074/075), Dharan, Nepal. Permission was also taken from the management division of Department of Health Services, Kathmandu (Ref: 179). The respondents were informed about the purpose of data collection. Informed consent, both written and verbal was taken from all the participants. The participants were assured about the confidentiality and they had full authority to accept or refuse to take part in the study.

## Results

Table 1 shows the socio-demographic characteristics of 270 respondents with 100% response rate. The mean age and standard deviation of respondents was 36.64±12.13. Most of the respondents (59.6%) were females. Educational status records of respondents show 25.9% had a middle school certificate followed by a high school certificate (23.7%). Majority 79.6% of respondents were married and 85.2% practiced Hinduism.

**Table 1:** Socio-demographic characteristics of respondents (n = 270).

Characteristics	Categories	Frequency (n = 270)	Percent (%)
**Age (years)**	<25	45	16.7
	25–35	86	31.8
	35–45	60	22.2
	≥45	79	29.3
**Mean age in years ± SD (Min-Max)**		36.64 ± 12.13 (18–76)	
**Sex**	Male	109	40.4
	Female	161	59.6
**Ethnicity**	Dalit	8	3.0
	Disadvantage Janajatis	67	24.8
	Disadvantaged non-dalitterai caste	14	5.2
	Relatively advantaged Janajatis	57	21.1
	Upper caste group	124	45.9
**Education**	Illiterate	38	14.1
	Primary school	34	12.6
	Middle school	70	25.9
	High school	64	23.7
	Intermediate	14	5.2
	Graduate or above	50	18.5
**Marital status**	Unmarried	47	17.4
	Married	215	79.6
	Others	8	3.0
**Religion**	**Hindu**	**230**	**85.2**
	Buddhist	25	9.3
	Christian	15	5.5
**Occupation**	Unemployed	14	5.2
	Business	60	22.2
	Private	37	13.7
	Government	16	5.9
	Labor	12	4.4
	Home maker	79	29.3
	Student	37	13.7
	Other	15	5.6
**Period of residence (near hospital)**	<5	66	24.4
	5–10	92	34.2
	10–15	50	18.5
	15–20	12	4.4
	≥20	50	18.5

### Knowledge and attitude of respondents

Knowledge was assessed based on 10 questions and classified as adequate and inadequate using mean percentage score. Similarly, attitude was assessed using 6 statements and classified as positive and negative based on mean percentage score. About 26.3% of community people had inadequate knowledge and 40.0% of the respondents had negative attitude towards healthcare waste.

### Risk perception towards healthcare waste

Risk perception was assessed based on 19 statements which were later dichotomized as good and poor based on mean score. More than half of respondents (52.2%) had poor risk perception towards healthcare waste as shown in Table 2.

**Table 2:** Risk perception towards healthcare waste (n = 270).

Statements	Strongly Agree	Agree	Neutral	Disagree	Strongly Disagree
All health care wastes are hazardous*	69 (25.6)	62 (23.0)	22 (8.1)	101 (37.4)	16 (5.9)
Liquid waste (blood and body fluid) is harmful	138 (51.1)	113 (41.9)	16 (5.9)	3 (1.1)	0 (0.0)
Children are more at risk as they may play with discarded syringe/needles	104 (38.5)	151 (55.9)	9 (3.3)	6 (2.3)	0 (0.0)
Throwing used cotton and gauges outside hospital is harmful	84 (31.1)	153 (56.7)	16 (5.9)	17 (6.3)	0 (0.0)
Recyclable products from HCW may not spread disease in the population	12 (4.5)	76 (28.1)	84 (31.1)	77 (28.5)	21 (7.8)
Sharps waste cannot be dangerous to human health*	7 (2.6)	11 (4.1)	9 (3.3)	131 (48.5)	112 (41.5)
Storing waste inside for longer period creates foul smell	137 (50.7)	127 (47.0)	2 (0.7)	3 (1.2)	1 (0.4)
Waste treatment leads to decrease in volume, weight and risk of infectivity	42 (15.6)	173 (64.1)	50 (18.5)	4 (1.4)	1 (0.4)
Animals like dogs visiting the disposal sites spread diseases to community	101 (37.4)	155 (57.4)	7 (2.6)	7 (2.6)	0 (0.0)
Hospital incinerator is one of the source of air pollution	78 (28.9)	124 (45.9)	54 (20.0)	13 (4.8)	1 (0.4)
Infectious waste may not transmit HIV/AIDS*	9 (3.3)	68 (25.2)	64 (23.7)	94 (34.8)	35 (13.0)
Infectious waste may transmit hepatitis B and C	32 (11.9)	105 (38.9)	109 (40.4)	19 (7.0)	5 (1.8)
Expired drugs can have negative health effects	115 (42.6)	125 (46.3)	21 (7.8)	7 (2.6)	2 (0.7)
Improperly managed health care waste may cause cancer in future	31 (11.5)	160 (59.3)	66 (24.4)	12 (4.4)	1 (0.4)
Improperly managed waste may contaminate water source	104 (38.5)	150 (55.6)	5 (1.9)	10 (3.6)	1 (0.4)
Improperly managed waste may not contaminate soil*	7 (2.6)	35 (13.0)	22 (8.1)	158 (58.5)	48 (17.8)
Healthcare wastes are generally mixed with solid waste	11 (4.1)	73 (27.0)	47 (17.4)	134 (49.6)	5 (1.9)
Mixing healthcare waste with solid waste is harmful	61 (22.6)	150 (55.6)	33 (12.2)	26 (9.6)	0 (0.0)
Residences nearby hospitals are suffering more health effects than others	73 (27.0)	160 (59.3)	14 (5.2)	22 (8.1)	1 (0.4)

Figure in parenthesis are in percentage

*Reverse statement

Bivariate analysis was carried out to determine the association between potential factors with risk perception. It was found that education (p = 0.001), year of residence near hospital (p = 0.026), health and other problems faced (p = 0.006) and knowledge (<0.001) were significant. However, binary logistic regression analysis indicated that knowledge independently influenced risk perception about healthcare waste management (Table 3). Respondents who had adequate knowledge regarding healthcare waste were 3.3 times more likely to have good risk perception compared to those who had inadequate knowledge. (AOR = 3.31, CI: 1.673–6.581; p = 0.001)

**Table 3:** Binary logistic regression analysis showing factors associated with risk perception.

Variables	Categories	β	Odds Ratio	95% CI for Odds Ratio		p value
				Lower	Upper	
Sex	Male	0.473	1.605	0.932	2.765	0.088
	Female	Ref				
Ethnicity	Upper caste	0.163	1.177	0.691	2.765	0.549
	Non upper caste	Ref				
Education	Literate	0.791	2.205	0.925	5.257	0.074
	Illiterate	Ref				
Residence near hospital	> 20 years	0.372	1.451	0.736	2.861	0.282
	≤ 20 years	Ref				
Health and other problems faced	Yes	0.458	1.581	0.833	3.002	0.161
	No	Ref				
Complained about hospital’s HCWM	Yes	0.122	1.130	0.581	2.465	0.759
	No	Ref				
Knowledge on HCWM	Adequate	1.199	3.318	1.673	6.581	0.001*
	Inadequate	Ref				
Constant		-1.373	0.253		0.004	

*Significant at p<0.*05 Ref*: *Reference*

## Discussion

Limited studies have been conducted on risk perception towards healthcare waste in the context of Nepal. In this study, about 52.0% of respondents had poor risk perception towards healthcare waste, 26.3% had inadequate knowledge and 60.0% had positive attitude towards healthcare waste management. A study done in Nigeria revealed that 12.0% respondents showed poor knowledge, good attitude and poor perception while 10.0% showed good knowledge and attitude but poor perception. [16]. In this study, 82.0% of respondents knew about necessity for waste segregation, but only 6.0% could correctly answer that segregation should be done at point of generation. A study done in Namibia revealed that 85.7% ward assistants and 90.0% of cleaners knew that health care wastes are hazardous and could pose health risks if not properly segregated. [17] A study in Pakistan showed that doctors and nurses have better knowledge than paramedics and sanitary workers about infectious waste management. [18] In contrary, 95.2% of respondents demonstrated good knowledge of hazardous healthcare waste in the done in Nigeria. [19]. Educational status might influence the differences.

This study showed that 60.0% and 65.0% of respondents respectively rated the hygiene of the hospital and the disposal system as average. The result is inconsistent with the study done in Ethiopia where 66.3% of participants replied very good hygiene inside the hospital compound and 45.6% replied well for the waste disposal system. [8] This might be due to the low priority given to hospitals regarding healthcare waste management in context of Nepal. [14]

More than half of respondents had poor risk perception towards healthcare waste in this study. A study showed medical doctors had better risk perception than other health workers. [4] The different findings might be due to the awareness level between general public and medical persons. A study done in Ethiopia revealed that people who identified healthcare wastes as a potential source of air pollution had a high perceived risk. [8] In the present study, respondents who had adequate knowledge regarding healthcare waste were 3.38 times more likely to have good risk perception compared to those who had inadequate knowledge. It is nearly similar to the study done in Ethiopia where those people who had knowledge about healthcare waste management had twice the risk of their counterparts. [8] The dumping of hazardous waste, chemicals, and landfills were seen as posing the highest risks to participants and their families in a study done by Brandi M et.al. [20]

In the present study, 28.0% of respondents who resided near the hospital admitted that they faced problems. Major complaints were malodor from the hospital, viral fevers, cough and cold (respiratory symptoms). In a similar study done in United Kingdom, an increased prevalence of symptoms such as fatigue, sleepiness and headaches were self-reported. [21]

### Limitations

A qualitative study would have been more informative to support the quantitative findings. The factors that influence risk perception about healthcare waste management among community members might differ across settings but this was not explored in the present study. The specific 1km radius was not measured in this study. However, this may be examined in future studies.

## Conclusion

The risk perception towards healthcare waste among the respondents was found to be poor. Half of the respondents were not aware of the risk associated with healthcare wastes. Knowledge was a strong predictor of risk perception towards healthcare waste among members of the community. This highlights the need for extensive awareness programs. Promoting knowledge about healthcare waste is a way to change the perception in Nepal. Massive utilization of social media, audio visual aids to disseminate the information to public on risk associated with healthcare waste is recommended. Community engaged research approach is needed to address environmental health concerns among public residents.

## Supporting information

S1 Data (XLSX)

## List of abbreviations

HCWM: Healthcare Waste Management
BPKIHS: B.P. Koirala Institute of Health Sciences
